# Magnetization reversal in YIG/GGG(111) nanoheterostructures grown by laser molecular beam epitaxy

**DOI:** 10.1080/14686996.2017.1316422

**Published:** 2017-05-18

**Authors:** Boris B. Krichevtsov, Sergei V. Gastev, Sergey M. Suturin, Vladimir V. Fedorov, Alexander M. Korovin, Viktor E. Bursian, Alexander G. Banshchikov, Mikhail P. Volkov, Masao Tabuchi, Nikolai S. Sokolov

**Affiliations:** ^a^ Divisions of Solid State Physics and Physics of Dielectrics and Semiconductors, Ioffe Physical-Technical Institute of Russian Academy of Sciences, St Petersburg, Russia; ^b^ Synchrotron Radiation Research Center, Nagoya University, Nagoya, Japan

**Keywords:** YIG nanolayers, magnetization process, magnetic anisotropy, laser MBE, XMCD, AFM, XRD, MOKE, 40 Optical, magnetic and electronic device materials, 102 Porous / Nanoporous / Nanostructured materials, 203 Magnetics / Spintronics / Superconductors, 306 Thin film / Coatings

## Abstract

Thin (4–20 nm) yttrium iron garnet (Y_3_Fe_5_O_12_, YIG) layers have been grown on gadolinium gallium garnet (Gd_3_Ga_5_O_12_, GGG) 111-oriented substrates by laser molecular beam epitaxy in 700–1000 °C growth temperature range. The layers were found to have atomically flat step-and-terrace surface morphology with step height of 1.8 Å characteristic for YIG(111) surface. As the growth temperature is increased from 700 to 1000 °C the terraces become wider and the growth gradually changes from layer by layer to step-flow regime. Crystal structure studied by electron and X-ray diffraction showed that YIG lattice is co-oriented and laterally pseudomorphic to GGG with small rhombohedral distortion present perpendicular to the surface. Measurements of magnetic moment, magneto-optical polar and longitudinal Kerr effect (MOKE), and X-ray magnetic circular dichroism (XMCD) were used for study of magnetization reversal for different orientations of magnetic field. These methods and ferromagnetic resonance studies have shown that in zero magnetic field magnetization lies in the film plane due to both shape and induced anisotropies. Vectorial MOKE studies have revealed the presence of an in-plane easy magnetization axis. In-plane magnetization reversal was shown to occur through combination of reversible rotation and abrupt irreversible magnetization jump, the latter caused by domain wall nucleation and propagation. The field at which the flip takes place depends on the angle between the applied magnetic field and the easy magnetization axis and can be described by the modified Stoner–Wohlfarth model taking into account magnetic field dependence of the domain wall energy. Magnetization curves of individual tetrahedral and octahedral magnetic Fe^3+^ sublattices were studied by XMCD.

## Graphical abstract

**Figure:**
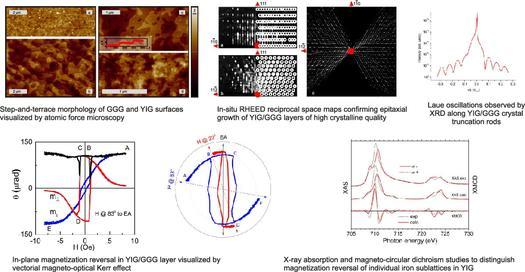

## 1. Introduction

Yttrium iron garnet (YIG) is a model object for study of magnetism in crystals. Its structural, electrical, magnetic, optical and acoustic properties have been intensively discussed since the middle of the last century [e.g.^1,2^]. The interest in YIG is related to its unique physical properties such as high optical transparency in a wide range of wavelengths, high Curie temperature, ultra-low spin wave and acoustic wave damping, and strong magneto-optical effects. In the 1970–1980s much attention was paid to fabrication of heterostructures with garnet layers because of the development of new magneto-optic [3,4] and microwave [5] devices as well as applications utilizing cylindrical domain control for data storage and processing [6–8].

Garnet films of micrometer thickness were routinely grown by liquid phase epitaxy. Extensive studies of static, dynamic, magnetic and magneto-optical properties of such films have been carried out [1,3,4,9]. It has been shown that magnetic anisotropy (in-plane, out of plane, rhombic) in thick YIG based heterostructures can be tailored by tuning the film/substrate composition and lattice parameters. The study of magnetization reversal and domain structure in garnet films showed that structurally and magnetically the interface differs from the bulk of the film. Although from crystallographic and magnetic points of view YIG and gadolinium gallium garnet (GGG) are centrosymmetric, near the interface the center of symmetry is lost, giving rise to optical second harmonic generation of crystallographic [10] and magnetic [11] types as well as to linear magnetoelectric effect [12,13].

The interest in YIG nanostructures, their fabrication and properties, has developed only recently, as new research fields, such as oxide spintronics, magnonics [14–16] and spin caloritronics [17], have branched off owing to developments in spintronics. Ultrathin magnetic insulating layers (e.g. YIG) may find applications in spintronics for data transmission in the form of spin wave packets [18]. This approach may provide certain advantages over spin-polarized electron currents or spin currents that can be induced in conductive media. Understanding YIG growth is also of great interest in studies of YIG/(Pt, Ta) metal-insulator heterostructures [19–25] for spin pumping and spin transfer, as well as for YIG growth on topological insulators [26].

The growth of thin high-quality epitaxial YIG layers has become possible upon development of laser molecular beam epitaxy (MBE). A number of recent works have addressed the growth of nanometer sized YIG layers on gallium gadolinium garnet GGG substrate. Investigations into structural, magneto-optical, static and dynamic magnetic properties of such layers have been reported [27–37]. It has been shown that magnetization in YIG layers is lower than in bulk crystals because the interface and surface regions are rich with defects and deficient in 3d bonds. Besides this, magnetic ordering different from bulk was observed near the interface [33]. The interfaces are supposed to be responsible for additional spin wave damping channels through magnon scattering on interface defects and through two-magnon decay. Nevertheless spin wave damping in such nanostructures may be low enough and comparable to values obtained for YIG single crystals [35–37], being a prerequisite for fabrication of novel effective spin wave devices.

It must be noted that up to now more attention has been paid to static and dynamic magnetic properties than to the magnetization reversal process. The latter is an important characteristic of a magnetic material and bears information not only about the magnetic interaction defining the change in magnetic state in applied magnetic field, but also about the structural quality of material. In YIG/GGG(111) structures grown by pulsed laser deposition the in-plane magnetization reversal is characterized by low coercivity (H_c_ ~ 1 Oe) hysteresis that may be accompanied by reversible magnetization rotation [34]. To the best of our knowledge this process has been studied only scarcely.

The aim of the present work is to grow YIG layers by laser MBE, to characterize their structural properties and to study mechanisms responsible for magnetization reversal in the in-plane and out-of-plane magnetic fields. The grown YIG/GGG(111) samples have been characterized by synchrotron X-ray diffraction (XRD), high-energy electron diffraction (RHEED) and atomic force microscopy (AFM). The magnetization reversal has been investigated using magneto-optical methods in X-ray and visible light range and by vibrating sample magnetometry (VSM). Magnetization dynamics was studied using ferromagnetic resonance spectroscopy.

## 2. Experimental details

YIG/GGG (111) heterostructures were grown by laser molecular beam epitaxy. The polished (111)-cut GGG substrates were annealed prior to growth for 3 h at 1000 °C in 0.5 mbar of oxygen to improve surface morphology. A stoichiometric Y_3_Fe_5_O_12_ target was ablated by 1 Hz × 10 ns pulses of KrF excimer laser (λ ≈ 248 nm) at a fluence of ~3.1 J cm^–2^. YIG layers were grown in 0.02 mbar of oxygen at substrate temperature of 700^–^1000 °C. The indicated temperatures were measured inside the stainless steel sample holder to which the sample was attached by silver paste glue. More than 20 YIG/GGG samples have been fabricated to understand the influence of growth parameters on YIG growth. In what follows we present data proved to be typical for particular growth regimes.

Crystal structure of YIG layers was investigated *in situ* by enhanced RHEED 3D mapping technique that allows measuring and visualizing three dimensional intensity distribution within a large reciprocal space volume, building arbitrary reciprocal space cross-sections/projections and performing comparison to model reciprocal lattices [38]. The 3D mapping was carried out by collecting series of RHEED patterns during sample rotation around the surface normal. More precise information on the crystal structure was obtained *ex situ* during synchrotron measurements at BL3A X-ray diffraction beamline (Photon Factory, Tsukuba, Japan). Intensity profiles along YIG/GGG (111) crystal truncation rods (CTR) were measured by point silicon drift detector as well as by applying the 3D mapping approach similar to the one described above. Dense series of XRD patterns were recorded with Pilatus 100 K detector sweeping the reciprocal space volume containing GGG and YIG CTR diffuse scattering around Bragg reflections and the background that has to be taken into account for accurate CTR modeling. Surface morphology of the grown layers was characterized by NT-MDT atomic force microscope in semi-contact mode.

**Figure 1.**
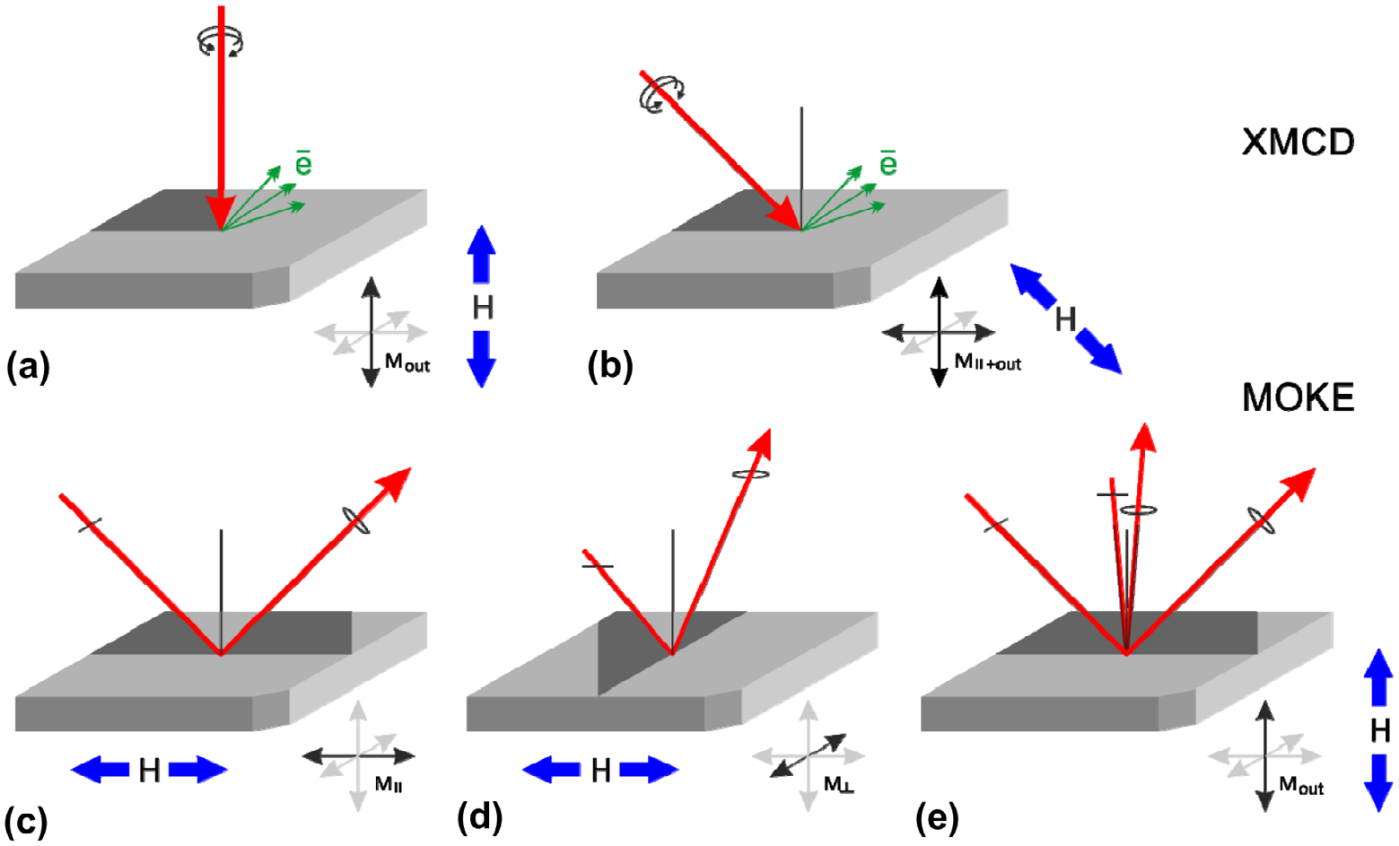
XMCD experiment geometries (a, b). MOKE experiment geometries: LMOKE_||_ (c) and LMOKE_⊥_ (d) for measuring in-plane magnetization components, PMOKE (e) for measuring out-of-plane magnetization components.

X-ray absorption (XAS) and X-ray magnetic circular dichroism (XMCD) measurements at Fe L edge were performed at BL16 beamline of Photon Factory synchrotron (Tsukuba, Japan). The measurements were carried out in surface sensitive total electron yield (TEY) mode keeping the sample biased at –12 V to decrease the influence of magnetic field on the measured electron current. XMCD measurements were carried out in magnetic fields of up to 12 kOe applied parallel to the beam direction. The beam was incident at 30 or 90° to the surface (Figure 1(a) and 1(b)). The TEY spectra were normalized to incident beam intensity and to the pre-edge intensity. The XMCD asymmetry signal at fixed energy and magnetic field was derived from the difference in absorption measured in σ+ and σ– circular polarized light (degree of circular polarization 96%). To increase sensitivity the incident light polarization was modulated at 10 Hz.

The in-plane magnetization curves were studied in the Quantum Design PPMS-9 system (Quantum Design Inc., San Diego, CA, USA) at room temperature. The net magnetic moment was calculated from magnetization taking into account the film thickness derived from RHEED and XRD data. Complementary to this, magnetization curves have been measured using vectorial magneto optical Kerr-effect set-up operating at λ = 405 nm. To gain sensitivity the incident light polarization was modulated with a quartz photo-elastic modulator (50 kHz) or a Faraday cell (400 Hz). In-plane magnetization components parallel (M_||_) and perpendicular (M_⊥_) to the applied in-plane magnetic field were measured using longitudinal magneto-optical Kerr effect by orienting the light incident plane parallel (LMOKE_||_) and perpendicular (LMOKE_⊥_) to the magnetic field direction (Figure 1(c) and 1(d)). In this set-up the light was incident at 45° to the surface in S-polarization geometry. The out-of-plane magnetization curves in magnetic fields of up to 20 kOe (4 kOe) were investigated by measured polar MOKE (PMOKE) effect at 87° (45°) incidence (Figure 1(e)). The beam was focused to a small spot 0.3 mm in diameter to allow precise area selective studies. Ferromagnetic resonance (FMR) spectra were measured on an electron paramagnetic resonance spectrometer at 9.41 GHz in magnetic field of up to 10 kOe applied in-plane or out-of-plane.

## 3. Surface morphology and lattice structure

### 3.1. Atomic force microscopy

**Figure 2.**
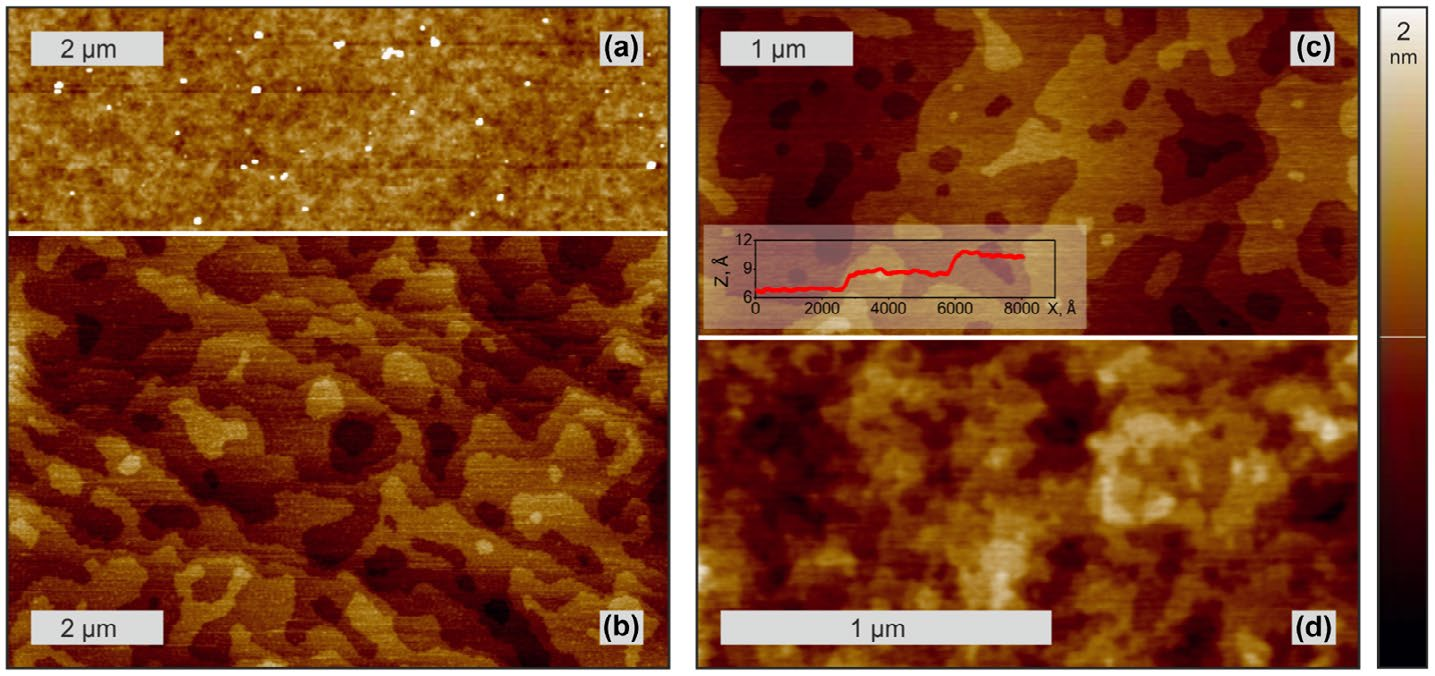
Typical surface morphology of a GGG substrate before (a) and after annealing (b) and of YIG layer grown at 1000 °C (c) and 850 °C (d). The inset shows a height profile across the two adjacent atomic steps.

The role of GGG substrate annealing prior to YIG growth becomes evident from analysis of AFM surface morphology images measured before and after 1000 °C annealing in oxygen (Figure 2(a) and 2(b)). The original substrate surface is rough (rms > 2.5 Å) showing traces of polishing. In contrast to this, the annealed surface shows atomically flat terraces separated by monoatomic steps (rms < 1.2 Å). The 1.8 Å step height agrees with the GGG lattice periodicity in [111] direction, which corresponds to 444 reciprocal lattice node. The curved terrace edges indicate that the mean free path of the atoms diffusing across the GGG surface during annealing stage is shorter than the vicinal surface terrace width (500–1000 nm) corresponding to the substrate miscut angle (0.01–0.02°).

Deposition of YIG at 800–1000 °C onto annealed GGG substrate was shown to produce a uniform YIG layer with atomically flat terraces and 1.8 Å atomic steps separating the terraces, in high resemblance to the surface morphology of annealed GGG substrates (rms < 1.2 Å) discussed above. AFM images presented in Figure 2(c) and 2(d) show such step-and-terrace morphology for the YIG layers grown at 1000 and 850 °C illustrating the dependence of characteristic terrace width on the growth temperature. The much increased adatom mobility makes the terrace width at 1000 °C (~500 nm, Figure 2(c)) about six times larger than the terrace width at 850 °C (~80 nm, Figure 2(d)). The surface morphology was found to be not dependent on the YIG layer thickness in the tested 4–20 nm range of layer thicknesses.

Interestingly YIG deposition at 1000 °C onto a non-annealed GGG surface results in a morphology similar to the one shown in Figure 2(b) and (c), meaning that high-temperature YIG growth heals the surface in a way similar to the one achieved by few hours of annealing. Though improving surface morphology by YIG growth is advantageous from the point of view of time consumption, the probable drawback of such technology is the introduction of a defect rich layer at the interface. An appropriate alternative tested by the authors is to heal the GGG substrate surface by GGG overgrowth at an elevated temperature (as will be reported elsewhere).

### 3.2. Reflection high energy electron diffraction

**Figure 3.**
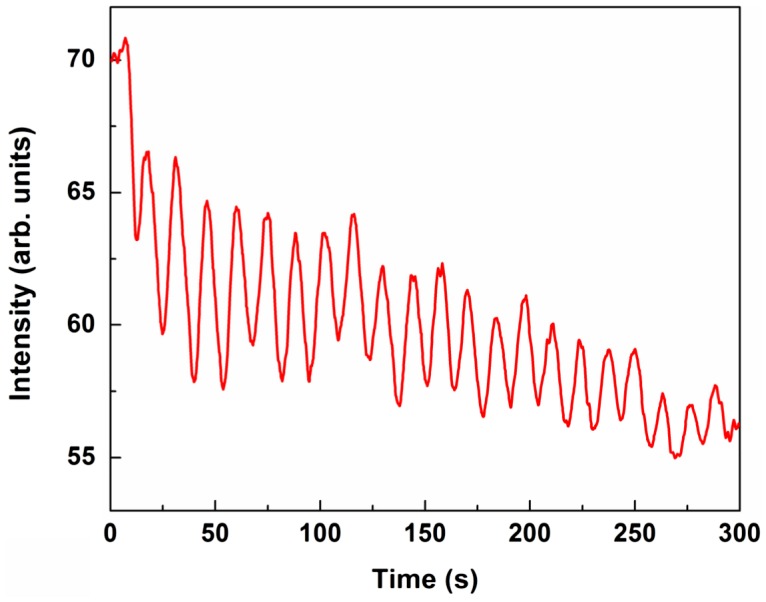
Typical RHEED specular beam intensity oscillations during YIG deposition at 700 °C.

The layer by layer growth regime characterized by low roughening rate was confirmed by observation of well-pronounced RHEED specular beam intensity oscillations (Figure 3) during YIG growth in the temperature range of 700–1000 °C. The oscillation period corresponds to the deposition of one YIG(111) monolayer (1 ML_111_=1.8 Å). The YIG growth rate within the studied sample series was calibrated to ~83 s nm^–1^ or ~15 s/ML_111_. The high precision control over the growth rate made it possible to keep the layer thickness constant within the series.

The intensity oscillations are known to visualize periodic modification of the growth surface morphology. The top layer is roughly half filled at intensity minimum and complete at intensity maximum. It happens often that oscillations are well pronounced at growth start and then damp gradually as the deposition proceeds further. This happens either due to surface roughening (the surface morphology is periodic in time locally however coherence over large areas is gradually lost) or because the growth switches to the step flow regime (in which the surface morphology almost stops changing, at least periodically). At YIG growth temperature of 700–850 °C slow damping layer by layer growth regime takes place with 20–30 detectable oscillation periods. Only 5–10 oscillation periods are observed for a growth temperature of 1000 °C, likely because the growth approaches the step flow regime with randomly curved steps due to increased adatom surface mobility.

**Figure 4.**
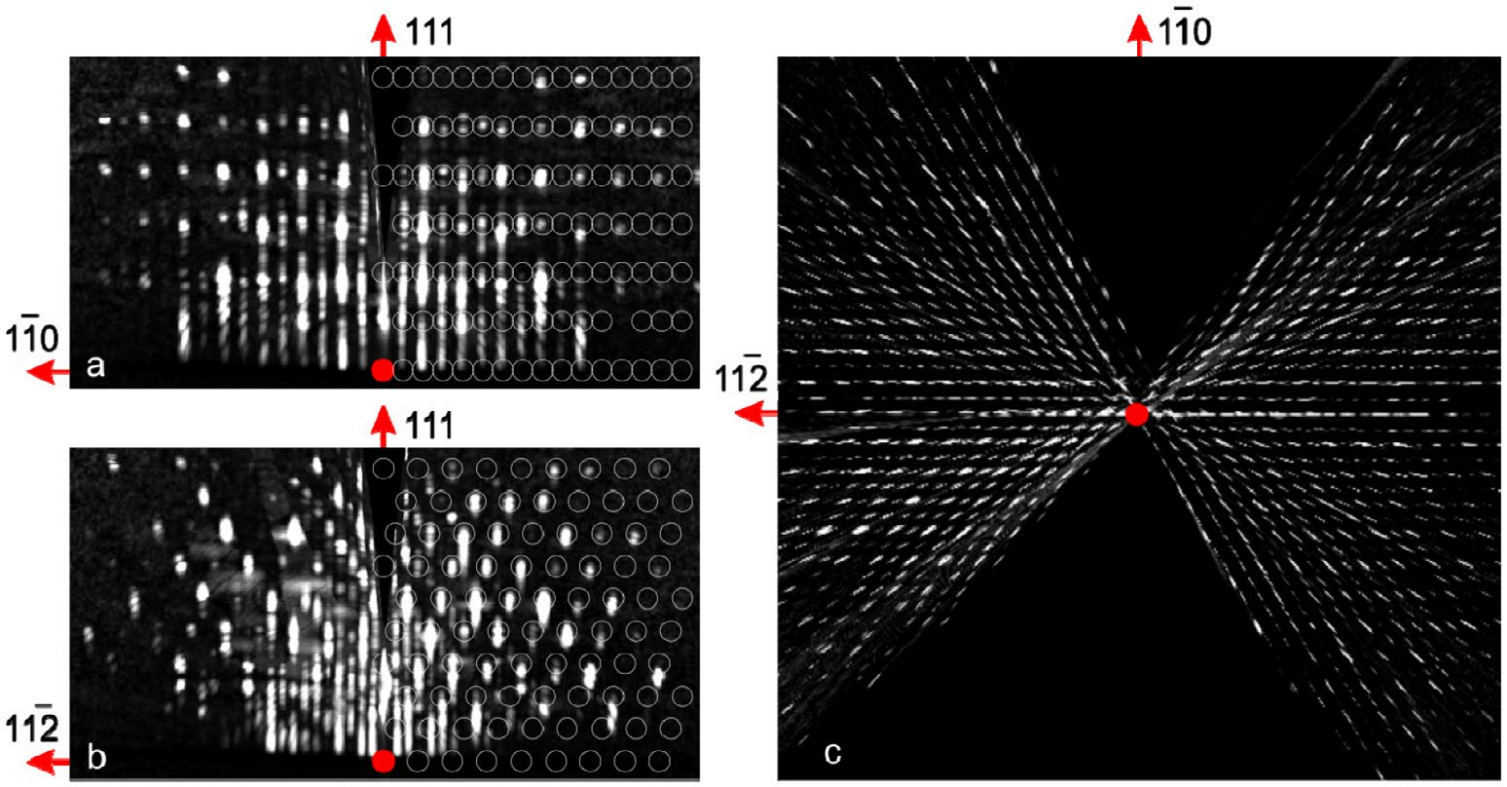
3D reciprocal space map measured by RHEED for a 10 nm YIG layer grown at 1000 °C. Reciprocal space side-views: [11-2] zone (a), [1-10] zone (b) and [111] plan view – projection onto the plane parallel to the (111) substrate surface (c). Superimposed are YIG reciprocal lattice nodes.

The first approach to crystal structure analysis of the grown YIG layers was carried out *in situ* by 3D mapping using electron diffraction. Figure 4 shows three orthogonal views of the 3D reciprocal space intensity distribution obtained typically for a YIG layer grown on GGG at 800–1000 °C. Figure 4(c) shows the plan view – a reciprocal space projection onto the plane parallel to the (111) substrate surface. The plan view shows a clear hexagonal pattern of crystal truncation rods confirming that the grown film is pure YIG with no undesired crystallites that are either non-stoichiometric (other forms of iron/yttrium oxides), textured or exhibiting different epitaxial relations. The latter claim refers to the full depth of the film as the RHEED intensity maps taken during film growth (not shown) were similar to the ones taken at the end of growth and presented in Figure 4. The crystallographic purity of the grown YIG layers was also confirmed by absence of the unindexed reflections on the long range *θ*–2*θ* XRD profiles (not shown).

Figure 4(a) and 4(b) show side views – cross-sections passing through reciprocal space origin (also known as zones) perpendicular to [11-2] and [1–10] directions. Comparing the experimentally obtained 3D intensity distribution to the model reciprocal lattice (shown in circles) it may be concluded that YIG lattice is co-oriented with that of GGG. The good agreement with model, the absence of additional unexplained reflections and small reflection size indicate that the grown YIG layer is of high crystalline quality. It is noteworthy that reflections with low out-of-plane component (low exit angle β) look elongated, indicating that the surface is considerably flat. The reflection out-of-plane width is reverse proportional to the diffraction volume thickness which for a flat surface is limited by electron escape depth proportional to sin(β). Therefore the reflections become progressively wider along the surface normal as the out-of-plane component (exit angle) becomes lower (Figure 4(a) and 4(b)). The shown model explains only the Bragg reflection positions and shape but not their intensity. The reflection intensities are defined by the YIG cell structure factor and are not so easy to be modelled for electron diffraction where the simple kinematical approach in most cases cannot be applied.

### 3.3. X-ray diffraction

**Figure 5.**
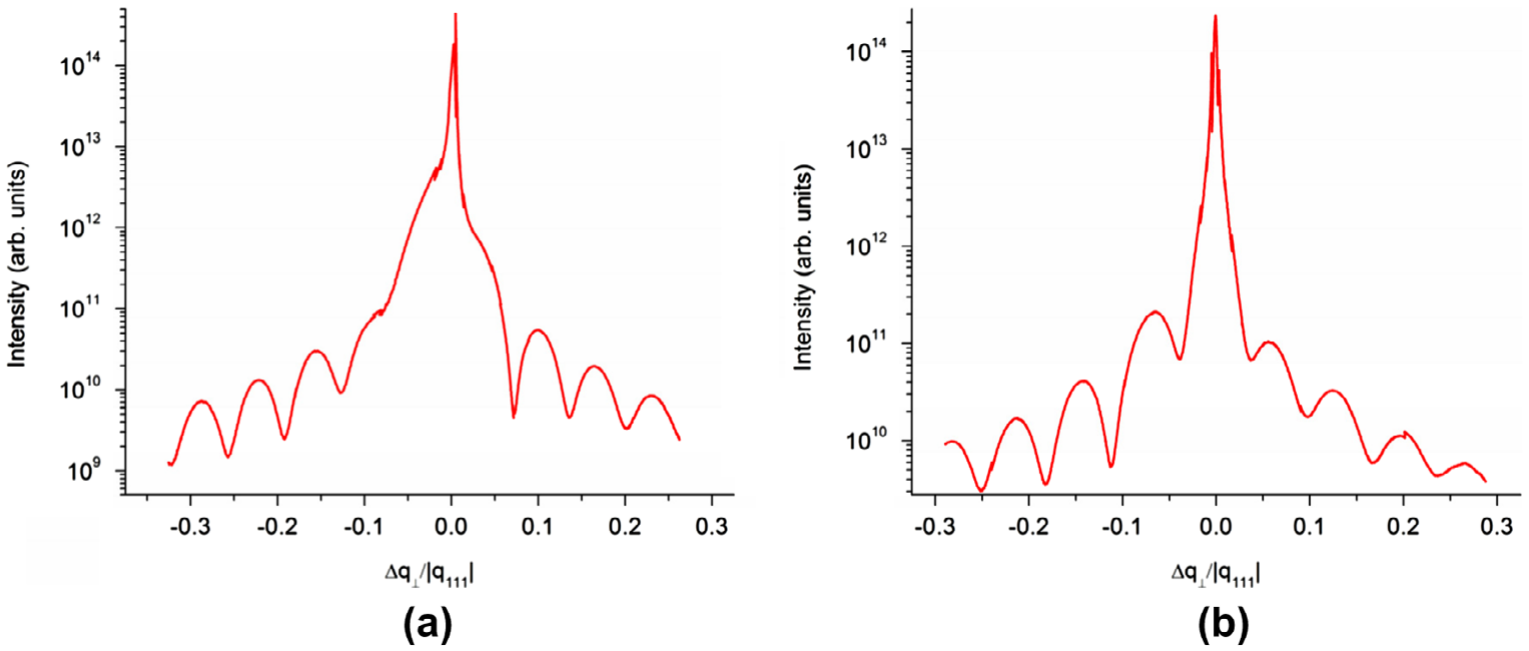
XRD intensity profiles through off-specular YIG/GGG (800) reflections measured along the [111] direction for 10 nm thick YIG layers grown at 850 °C (a) and 1000 °C (b). The profiles are plotted as a function of the perpendicular momentum transfer relative to the GGG (800) reflection. The q units are conveniently set to correspond to reciprocal of the distance between the GGG (111) layers.

Synchrotron XRD measurements of specular and non-specular crystal truncation rods have been carried out to analyze the YIG layer crystal structure, interface sharpness and exact film thickness. Figure 5 shows [111] intensity profiles passing through non-specular YIG(800) and GGG(800) reflections for two 10 nm YIG layers grown at 850 and 1000 °C. It has been confirmed by mapping intensity in the volume around the YIG/GGG CTRs that within the experimental accuracy the YIG and GGG rods coincide laterally. Thus it can be claimed that the YIG layer grows pseudomorphic to the GGG substrate. In the direction normal to the surface YIG/GGG (800) peaks are slightly shifted with respect to each other. For bulk materials the YIG/GGG lattice mismatch is very low (a_GGG_ = 12.383 Å, a_YIG_ = 12.376 Å, ∆a/a = 6·10^−4^) and the Bragg reflections are expected to considerably overlap. Nevertheless in the measured intensity profiles the YIG film contribution may be readily identified as the YIG Bragg peak is 20 times wider than that of GGG and shows well pronounced Laue oscillations. The oscillation period provides an accurate measure of the YIG layer thickness, the 10 ± 1 nm value being in good agreement with the estimation carried out by monitoring RHEED specular beam intensity during growth.

The experimentally observed out-of-plane shift between YIG and GGG reflections is larger than expected for bulk materials. To measure this shift with high accuracy the intensity distribution along the CTRs was modeled within the kinematic approach. It has been shown that for the YIG layers grown at 700–850 °C the lattice is stretched along the [111] axis by Δa_111_/a_111_ ≈ 8·10^−3^. This is slightly lower than the 1.2·10^−2^ distortion reported in our previous study,[34] where 10 nm YIG layers were grown at 650 °C in 7·10^−3^ mbar of oxygen. The crystal lattice of the layers grown at 1000 °C is deformed much less (Δa_111_/a_111_ < 4·10^−4^). The lower deformation tendency was confirmed to be present in the samples with twice higher 1000 °C layer thickness as well as in the samples in which 1000 °C YIG layer was grown on a few nm 700 °C seeding layer [39]. The observed rhombohedral lattice distortion is likely related to the defect structure of the layers (oxygen and iron vacancies, probable Ga diffusion from the substrate, etc.). Despite the distortion the interface with GGG is sharp enough as evidenced by high contrast and slow damping rate of the Laue oscillations. Worth noting is the difference in oscillation contrast observed to the left and to the right of the YIG/GGG Bragg peak. This difference is systematically present in our YIG/GGG samples and is likely due to some minor gradient of the (111) interlayer distance. A detailed study of this phenomenon is underway and will be presented elsewhere.

## 4. Magnetic anisotropy and mechanisms of magnetization reversal: vibrational and optical magnetometry

The following section will address the study of magnetic anisotropy and magnetization reversal in the grown YIG/GGG layers. It has been found that though the film thickness has some influence on magnetic properties (such as saturation and effective magnetizations) it has no fundamental effect on the magnetization switching behavior of these films. Although in what follows the results of magnetic studies will be shown for either 10 nm or 20 nm film (depending on the data availability and quality) we claim that the conclusions related to the magnetization switching are valid for the whole 10–20 nm thickness range.

**Figure 6.**
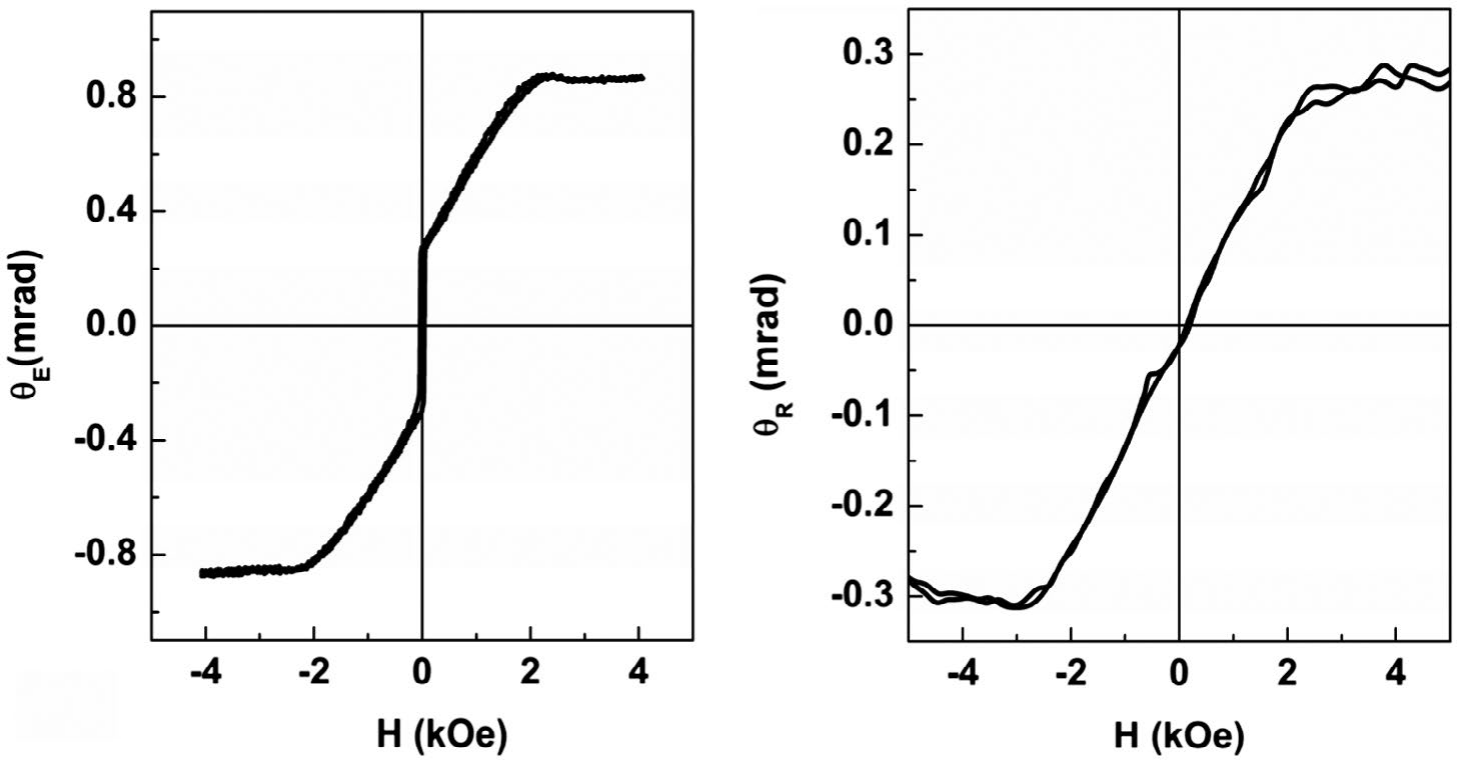
Magnetic field dependence of reflected light ellipticity *θ*_E_ at 45° incidence (a) and polarization rotation *θ*_R_ at normal incidence (b) for a 20 nm YIG film grown at 800 °C. Magnetic field is perpendicular to the film plane.

A typical magneto-optical response of the YIG film to the magnetic field applied perpendicular to the film plane is shown in Figure 6. Two complementary magneto-optical experiments were carried out in two different MOKE setups: measuring ellipticity at 45° incidence and measuring polarization plane rotation at almost normal incidence. The reflected light ellipticity *θ*_E_ measured at 45° incidence (Figure 6(a)) is simultaneously influenced by PMOKE (proportional to the ***m***_out_ magnetization component) and LMOKE (proportional to the ***m***_in_ magnetization component). The observed ellipticity jump at zero field is the LMOKE response to ***m***_in_ switching caused by the small non-zero in-plane magnetic field (due to slight deflection of the field direction from the film plane normal). The reflected light polarization rotation *θ*_R_ measured at normal incidence (Figure 6(b)) shows no jumps as it is related exclusively to the PMOKE which is proportional to the *m*_out_ magnetization component. The magnetization curves are reversible indicating that the magnetization switching occurs via rotation. The saturation magnetic field H_s_ ≈ (2–3) kOe in the studied samples is higher than the value of 4πM_s_ = 1.75 kOe in bulk YIG and YIG nanofilms 4πM_s_ = (1.1–1.5) kOe. This indicates the presence of an additional in-plane anisotropy field H_a_ ≈ –1 kOe which brings the magnetization in-plane in the absence of magnetic field. The presence of anisotropy field H_a_ is confirmed by FMR spectra measured both in parallel and perpendicular magnetic fields. Typical FMR curves and the calculated magnetic parameters of YIG films are given in [40].

**Figure 7.**
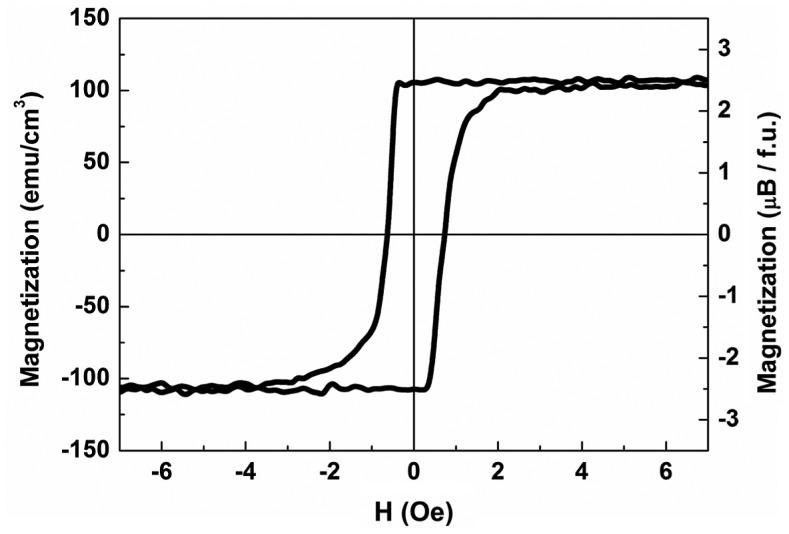
In-plane VSM magnetization curve measured for 20 nm YIG layer grown at 800 °C. Right vertical scale shows YIG magnetization per formula unit.

The in-plane magnetization curves have been measured both by VSM and MOKE. Figure 7 shows a VSM magnetization curve obtained for a 20 nm YIG film grown at 800 °C. The linear paramagnetic contribution of GGG substrate is properly subtracted. Very small coercive filed values below 2 Oe typical for the YIG/GGG samples studied in the present work are in good agreement with earlier reports [34]. The absolute value of saturation magnetization (4πM_s_ ≈1350 G) for the shown YIG film is noticeably lower than 4πM_s_ ≈1750 G reported for the bulk YIG crystals [2] but is in agreement with 4πM_s_ ≈ 1100–1500 G values reported in our earlier studies of YIG films grown at 650 °C [34]. The decreased magnetization in YIG layers may be caused by enhanced defect density and decreased number of 3d bonds at the upper and lower interfaces.

**Figure 8.**
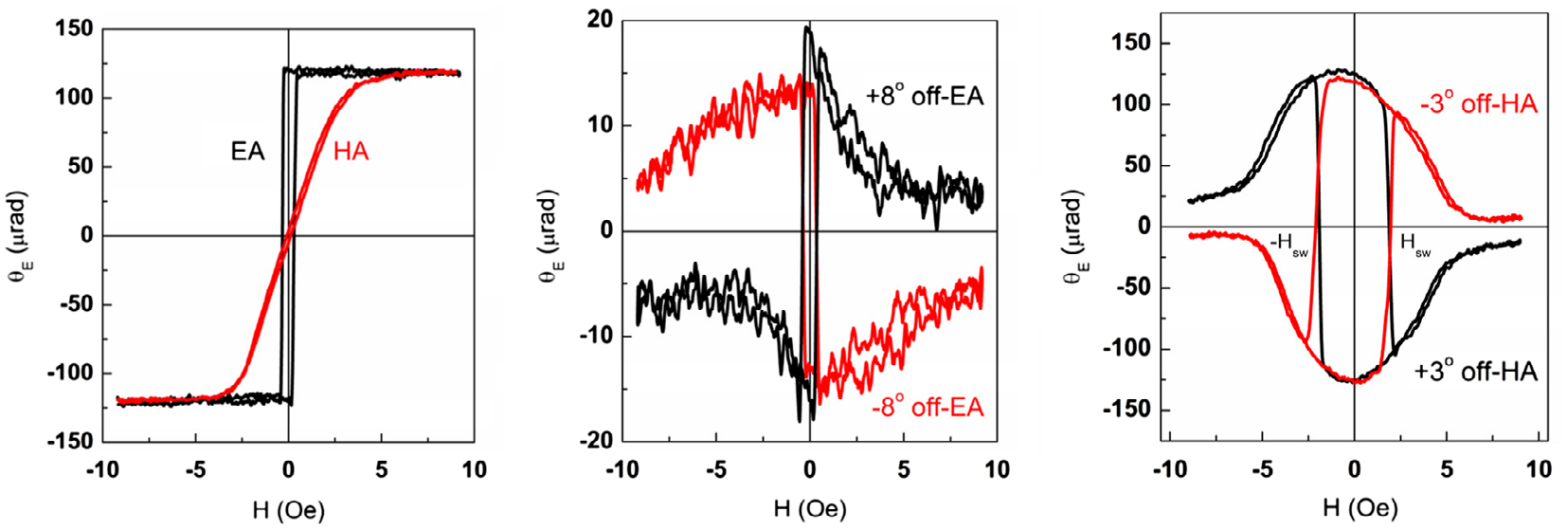
In-plane LMOKE field dependences: proportional to m_||_ for magnetic field oriented at 0° and 90° to the easy axis (a), proportional to m_⊥_ for magnetic field oriented at ±8° to the easy axis (b) and at ±3° to the hard axis (c).

Figure 8(a) shows in-plane LMOKE_**||**_ magnetization loops measured with incident light plane parallel to the applied magnetic field (see Figure 1(c)). In this geometry the magnetization component **m**_**||**_ parallel to the field is probed by measuring the reflected light ellipticity. The studied samples typically show very narrow hysteresis loops with coercive field H_c_ < 2 Oe in agreement with VSM results presented in Figure 7. The loop shape was shown to strongly depend on the orientation of magnetic field within the film plane, indicating the presence of an easy magnetization axis (EA) (compare EA and HA curves measured along and perpendicular to the easy axis).

In the YIG/GGG samples studied in [34] the **m**_**||**_ magnetization curve shape was slightly dependent on the magnetic field azimuth showing only weak coercive field anisotropy. In the samples discussed in the present work not only coercive field but also the overall shape of the **m**_**||**_ magnetization curve is highly dependent on the field azimuth. From the crystallographic point of view such in-plane anisotropy will not be present in YIG(111) layers. Most likely it appears due to some growth specific anisotropy factors such as small misorientation of the film surface normal with respect to the [111] direction, slightly off-normal plasma plume orientation, or uniaxial in-plane strain due to growth temperature gradient.

Figure 8(b) and 8(c) show in-plane LMOKE_⊥_ magnetization loops measured with incident light plane perpendicular to the applied magnetic field (see Figure 1(d)). In this geometry the magnetization component **m**_⊥_ perpendicular to the field is probed. The **m**_⊥_(H) dependence similarly to **m**_||_(H) dependence is strongly influenced by the magnetic field azimuth. The magnetization jumps are observed at field H = H_sw_, which is in general not coincident with H_c_. When the applied field is oriented close to the easy magnetization axis, the **m**_⊥_ component jumps simultaneously with **m**_**||**_ at H=H_c_ (Figure 8(b)). When the magnetic field is oriented close to the hard axis (HA) (Figure 8(c)) the **m**_⊥_ jump occurs at the magnetic field H_sw_, considerably different from the coercive field H_c_.

**Figure 9.**
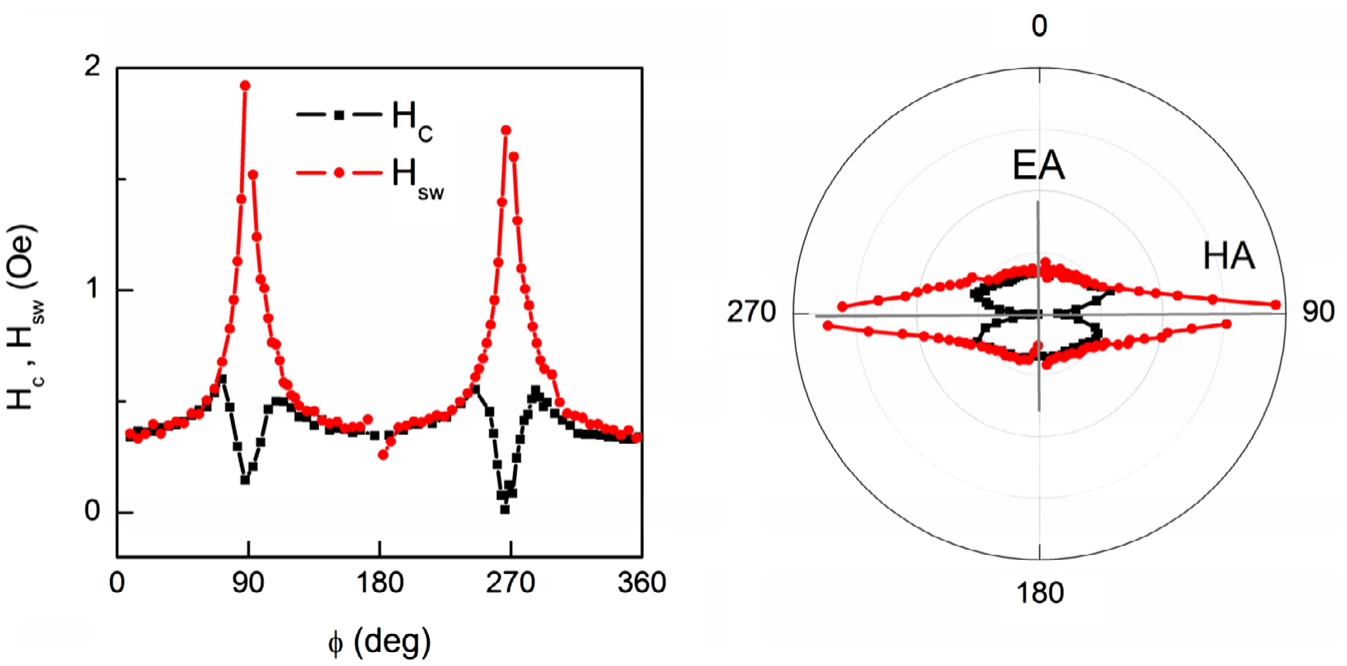
Angle variations of coercive field H_c_ and switching field H_sw_ in Cartesian and polar coordinates.

Fields H_c_ and H_sw_ were shown to depend drastically on the field azimuth φ (angle between magnetic field H_in_ and EA) (Figure 9) exhibiting a 180° rotation symmetry in agreement with the presence of the easy axis. Minimal value of H_c_ and maximal value of H_sw_ are observed for magnetic field oriented perpendicular to the easy axis.

**Figure 10.**
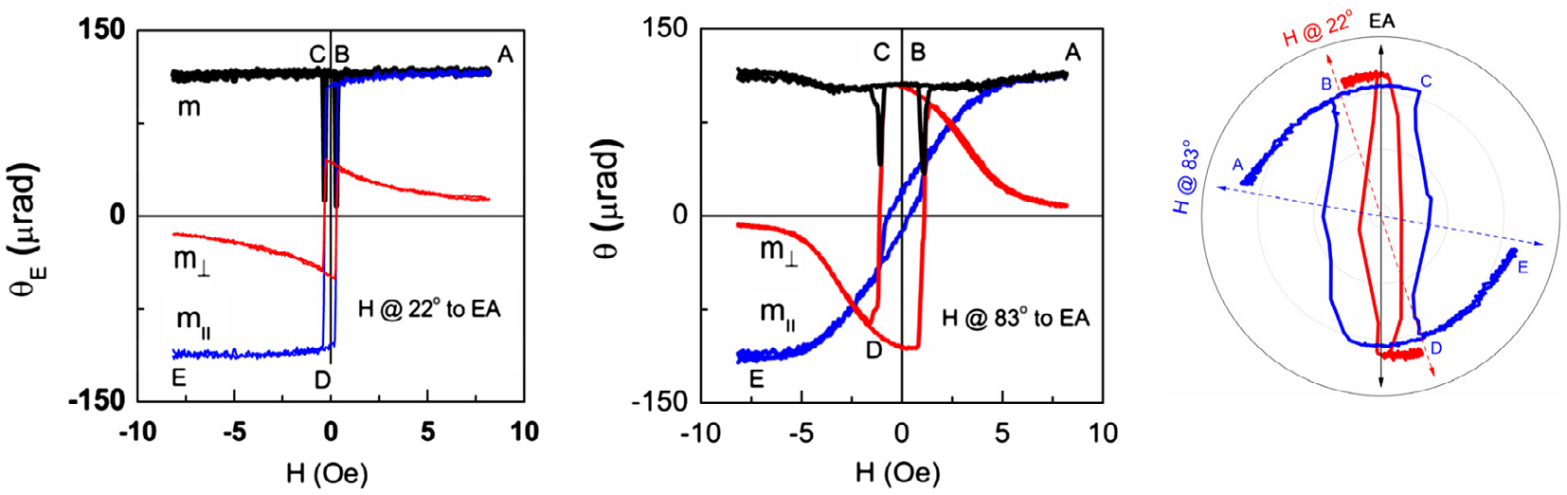
Magnetic field dependence of magnetization components m_⊥,_ m_||_ and m obtained from LMOKE measurements and magnetization vector trajectory for magnetic field oriented at 22° (a) and 83° (b) to EA.

Knowing m_||_ and m_⊥_ components one can plot the trajectory of the total magnetization vector **m** as a function of applied field H. Such vector representation of the magnetization reversal process in a YIG film is shown in Figure 10 for two magnetic field azimuths. In positive saturation the magnetization is practically parallel to the magnetic field. As the field is decreased, the magnetization vector gradually rotates (keeping the module constant), passes the easy axis at H = 0 and goes slightly beyond the easy axis until an abrupt magnetization jump takes place at H = −H_sw_. During the jump the magnetization module starts decreasing (nucleation of negative domains), goes down to almost zero (domain wall propagation makes the volumes of the opposite domains equal), and then recovers to its initial value (only negative domains remain). After the jump, the magnetization gradually rotates until it becomes parallel to the magnetic field at negative saturation. Figure 10 shows two magnetization curves obtained for magnetic field at 22° and 83° to the easy axis. As the magnetic field direction approaches the hard axis the switching field becomes higher and the magnetization becomes stronger deflected past the easy axis.

In principle such behavior (magnetization rotation plus non-reversible magnetization jumps) can be described within the classical Stoner–Wohlfarth model [41]. The model considers a single domain system with a uniaxial magnetic anisotropy in which magnetization is free to rotate but domain nucleation is prohibited. Such a system can be described by unitless potential W:(1)
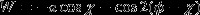



**Figure 11.**
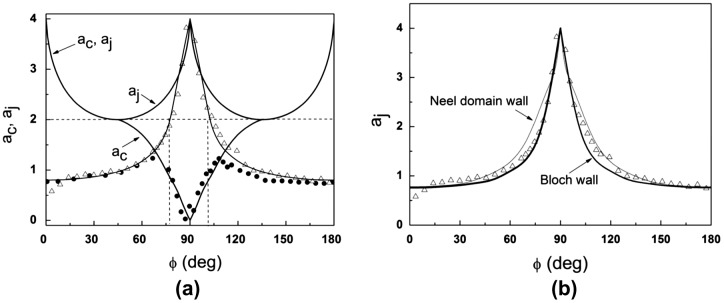
(a) Angle variations of parameters a_c_ and a_j_ in Stoner–Wohlfarth model (lines). Symbols correspond to experimental values of a_c_ and a_j_ obtained for a 10 nm YIG film. (b) Experimental (symbols) and calculated (lines) angle variations of parameter a_j_ from [41] for Bloch and Neel domain walls.

Here the unitless parameter a = 2·M_s_·H/K, M_s_ is the saturation magnetization, and K is the uniaxial anisotropy parameter. Angles φ and χ describe the easy axis and magnetization azimuths measured relative to the magnetic field direction. In this model the steady states of magnetization, switching fields and coercive fields are determined by W potential minima. Angle variations of unitless coercive field a_c_ = H_c_/H_a1_ (H_a1_ = 2·K/M_s_) and switching field a_j_ = H_sw_/H_a1_ are shown in Figure 11. Also shown in Figure 11 are experimental a_c_ and a_j_ values derived from hysteresis loops measured for a 10 nm YIG film. The anisotropy field value H_a1_ was estimated from m_||_(H) dependence measured for magnetic field oriented along HA (Figure 8(a)).

It becomes clear that in our system the classical Stoner–Wohlfarth model is applicable only in a narrow region of magnetic field azimuths (±10° off the hard axis) where magnetization reversal is determined mainly by magnetization rotation. Outside this region the magnetization jump takes place at fields much smaller than predicted by the classical theory. This happens because the magnetization jump in real samples is related to nucleation and movement of domain walls rather than to non-reversible magnetization rotation. Because the classical model is rarely realized, several modifications have been proposed in the literature allowing expanding application of the Stoner–Wohlfarth model to the cases where switching is followed by emergence of non-uniform magnetic structures [42–44]. Figure 11(b) shows the experimental angle variations of a_j_ parameter together with theoretical calculations carried out in [42]. In [42] the switching field was calculated for the case of pinning-depinning mechanism taking into account the magnetic field dependence of domain wall energy. We see that experimental dependence a_j_(*θ*) is in agreement with calculations for both Bloch and Neel domain walls. Note that because of small film thickness, in-plane magnetization and easy axis orientation, the Neel domain walls should be energetically more preferable as compared to the Bloch walls in spite of the fact that the energy density of Neel walls is higher than that of the Bloch ones.

**Figure 12.**
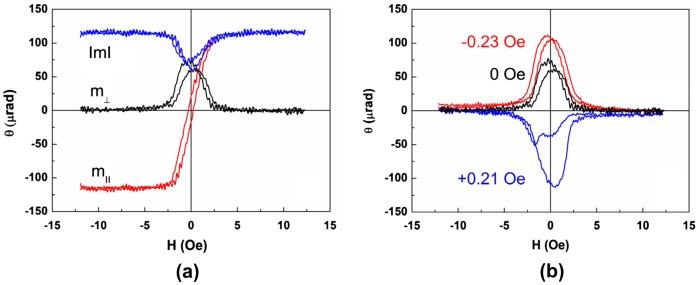
LMOKE magnetic field dependences in case when magnetic field is perpendicular to EA. Shown are m_⊥_, m_||_ and │m│ components for H_bias_ = 0 (a) and m_⊥_ component for bias field H_bias_ = +0.21 Oe, 0 Oe, –0.23 Oe, applied along EA (b).

Because the uniaxial in-plane magnetic anisotropy is rather small (H_a1_ ≤ 4 Oe) and the corresponding hysteresis loops are narrow, very small external fields (e.g. Earth’s magnetic field) can influence the magnetization reversal process especially when the applied magnetic field is orthogonal to the easy axis. Figure 12 shows m_⊥_ magnetization curves measured with sweeping magnetic field oriented 1° off the hard axis. Unexpectedly both hysteresis loop branches of m_⊥_ component are of the same sign which means that magnetization is rotated in the same direction. This indicates that a small constant magnetic field is present perpendicular to the HA. To estimate the value of this field, a bias constant magnetic field H_bias_ was applied in-plane perpendicular to the HA. As shown in Figure 12 with H_bias_ = 0 and 0.23 Oe the m_⊥_ magnetization curve is all positive while at H_bias_ = −0.21 Oe it becomes all negative. Thus the additional perpendicular field is of the order of 0.1 Oe which is somewhat lower than the magnetic field of Earth.

The in-plane easy magnetization axis in YIG/GGG(111) layers may appear due to several reasons. Among these are: substrate surface misorientation with respect to the (111) plane; parasitic magnetic field produced by the sample heater during growth; and in-plane uniaxial deformation due to minor temperature gradients at the growth or cooling down stages. In the studied YIG/GGG samples the easy axis orientation was shown to vary slowly by as much as a few tens of degrees across the sample surface. This rules out the substrate miscut and the parasitic magnetic fields as possible causes. Most likely the easy axis appears due to a very small in-plane YIG layer deformation. Deformation of the order of ▵a/a ~ 10^−5^ might be sufficient to account for an anisotropy field of 2–4 Oe. Distributed across the substrate surface a deformation as small as this is almost undetectable by X-ray diffraction or by another structural method. In lower quality YIG layers the characteristic lateral size of the uniformly deformed regions might be smaller than the spot size probed by MOKE. Thus the deformation effect could be eliminated upon averaging. In higher quality YIG layers averaging does not take place because the deformation variation has larger characteristic distance. Thus the easy axis variation becomes evident in **m**_⊥_(H) and **m**_**||**_(H) measurements.

## 5. X-ray absorption and magnetic circular dichroism

**Figure 13.**
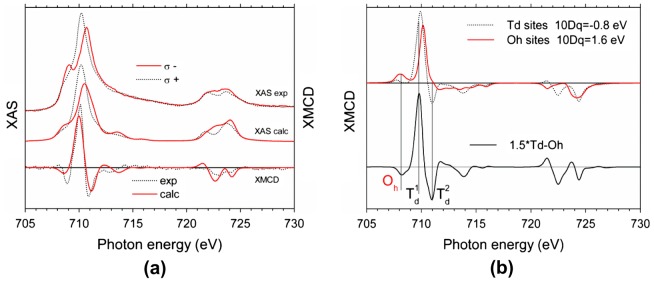
Experimental and calculated XAS/XMCD spectra for a 10 nm YIG layer in H = 6 kOe (a). Calculated XMCD spectra for Fe^3+^ ions in tetrahedral and octahedral positions as well as total XMCD signal (b).

While MOKE and VSM provide information about total magnetization, XMCD makes it possible to study magnetization of individual magnetic sublattices. Figure 13(a) shows typical XAS and XMCD spectra of a 10 nm YIG film grown at 1000 °C. The magnetic field of 6 kOe was applied perpendicularly to the film plane. The main features of XMCD spectrum are: strong positive maximum at 710 eV, two negative minima at 708.9 eV and 711 eV, and a mostly negative structure in the 720–725 eV energy region.

The fine structure of X-ray absorption and circular magnetic dichroism spectra at iron L-edge is caused by splitting of initial (2p^6^3d^5^) and final (2p^5^3d^6^) states [45,46] due to Coulomb interaction between 3d-electrons, spin-orbit coupling, ligand crystal field, and effects related to the appearance of holes on the 2p-level.

In YIG Fe^3+^ ions are known to occupy tetrahedral (24d) and octahedral (16a) positions forming two anti-parallel magnetic sublattices. The number of Fe^3+^ ions in tetrahedrons is 1.5 times higher than that in octahedrons, resulting in total YIG magnetization 4πM_s_ = 1750 G at room temperature. The crystal field characterized by parameter 10Dq is of opposite sign for 24d and 16a positions having values of 10 Dq = −0.8 eV for T_d_ iron sites and 10 Dq = 1.6 eV for O_h_ iron sites [47]. As a result the L_3_ absorption edge exhibits a fine structure consisting of two peaks analogous to the case of γ iron oxide [48].

To analyze XAS and XMCD spectra resolving contributions of the two different magnetic sublattices, numerical simulations have been carried out with the use of CTM4XAS software. Modeling within the frame of atomic multiplet theory accounting for the ligand crystal field [45,46,48,49] has been carried out, assuming Lorenzian and Gaussian broadening of 0.3 eV, and setting Slater–Condon parameters to 70% of the free ion values.

Figure 13(b) shows calculated XMCD spectra of individual sublattices and the total resultant XMCD spectrum of YIG layer being the appropriate sum of T_d_ and O_h_ contributions. Besides the main maximum labeled Td(#1) there are two minima noted as Oh and Td(#2), which can be identified as contributions of octahedral and tetrahedral magnetic sublattices. Having very similar characteristic features the calculated spectra are thus in good agreement with those observed experimentally (Figure 13(a)). The slight difference may be attributed to the charge transfer effects [48] as well as to nonlinearity of the measured TEY signal due to sample charging and saturation effects occurring at high X-ray intensity.

**Figure14.**
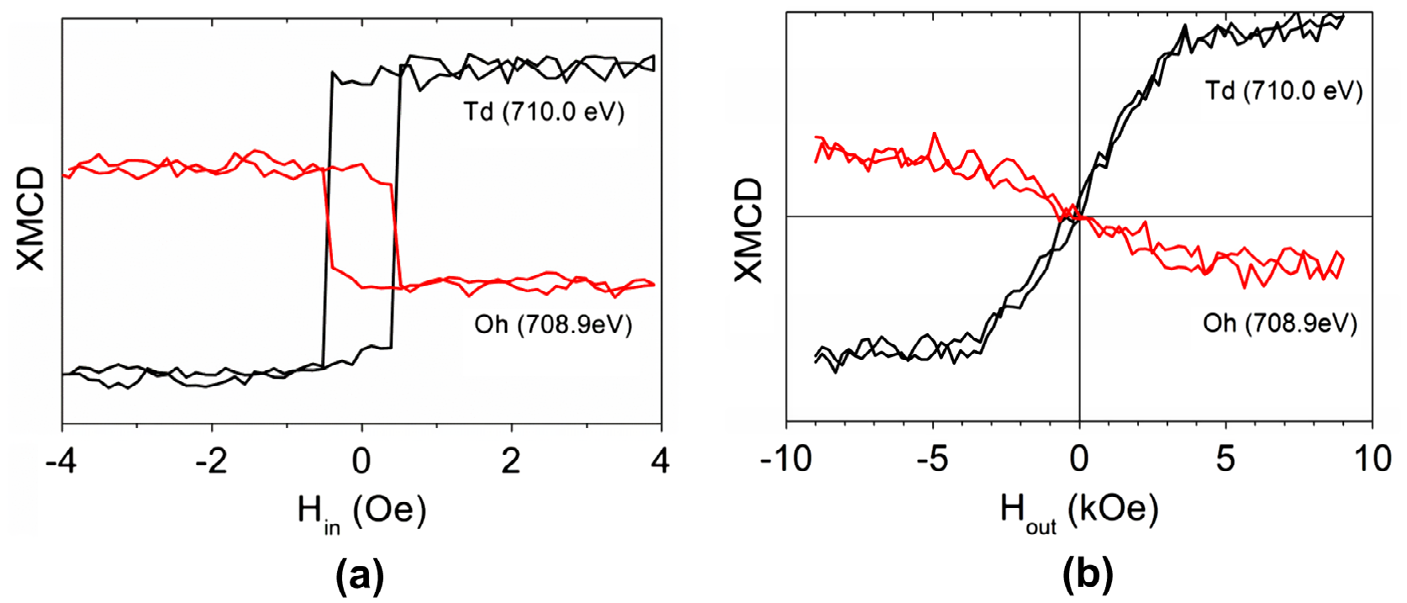
XMCD magnetization curves measured at E = 710 eV and 708.9 eV for magnetic field oriented 60° (a) and 0° off-normal (b).

It is clear from the modeled spectra that by choosing the appropriate photon energy one can make the XMCD measurement more sensitive to the magnetization of individual sublattice. At E = 708.9 eV XMCD signal is mostly proportional to the octahedral sublattice magnetization while at E = 710 eV XMCD probes the sum of octahedral and tetrahedral sublattice magnetizations with main contribution of the tetrahedral one. Magnetization curves measured at these two energies for magnetic field oriented 60° and 0° off-normal (see Figure 1 for particular geometry) are shown in Figure 14.

As expected the magnetization loops at 708.9 eV and 710 eV are of opposite sign. The in-plane magnetization curves show very narrow hysteresis loop in agreement with the results of vector MOKE magnetometry. The out-of-plane magnetization curve shows no hysteresis and reaches saturation H_out_ ~ 3 kOe (Figure 14(b)) in rough agreement with the polar Kerr effect measurements presented in Figure 6. Up to maximal fields H_out_ ~ 12 kOe the ratio between XMCD signals from Fe^3+^ ions in octahedral and tetrahedral positions remains constant being the evidence of that the corresponding magnetizations are anti-parallel.

## 6. Conclusions

AFM, XRD and RHEED studies of nanometer thick YIG(111) films fabricated by laser MBE on GGG(111) substrates in 700–1000 °C range of growth temperatures reveal that these films are of high crystalline quality, exhibit uniform thickness and sharp interfaces. The YIG layers show surface morphology consisting of atomically flat terraces with terrace width dependent on the growth temperature. The YIG lattice is pseudomorphic to GGG laterally and tends to become stretched in the normal direction as the growth temperature is decreased.

The range of growth conditions probed in the present work was found to mainly influence the surface morphology and (111) interplane distance. No drastic dependence of magnetic properties on growth conditions has been noticed.

Investigations of magnetic properties of the films show the presence of induced magnetic anisotropies. In absence of magnetic field the magnetization is oriented in-plane due to shape anisotropy and additional induced magnetic anisotropy field H_a_ ~ –1KOe analogously to films prepared by laser deposition. The study of in-plane magnetization reversal clearly demonstrates the presence of a small uniaxial in-plane magnetic anisotropy. Because the in-plane magnetic anisotropy is very low H_a1_ ~ (2–4) Oe it becomes sensitive to changes of the crystal structure, presence of strains, defects and so on. This makes the study of the in-plane hysteresis loops a very sensitive instrument for estimations of film quality and detecting small in-plane deformations, which cannot be detected by other methods. The in-plane magnetization reversal is followed by reversible magnetization rotation and non-reversible magnetization jump associated with nucleation and movement of domain wall. The process can be adequately described by the modified Stoner–Wohlfarth model which takes into account the dependence of domain wall energy on the applied magnetic field. Investigation of XAS and XMCD spectra of YIG films show that at definite photon energies XMCD is sublattice selective and can be used to study magnetization of tetrahedrally and octahedrally coordinated Fe^3+^ ions separately.

## Disclosure statement

No potential conflict of interest was reported by the authors.

## Funding

The work has received support from Russian Foundation for Basic Research [grant number 16-02-00410 A]. The synchrotron measurements were carried out along the proposals 2014G725 and 2014G726 at Photon Factory, Tsukuba, Japan. The work at the Ioffe Physical-Technical Institute was supported by the Government of the Russian Federation through the Program P220 [project number 14.B25.31.0025].

## Supplemental data

Supplemental data for this article can be accessed here. [http://dx.doi.org/10.1080/14686996.2017.1316422]

## Supplementary Material

Suppl.pdf
