# Supplementary material for: Magnetization reversal in YIG/GGG(111) nanoheterostructures grown by laser molecular beam epitaxy
Source: Sci Technol Adv Mater. 2017 May 18;18(1):351–63. doi: 10.1080/14686996.2017.1316422 (PMC5481691; doi:10.1080/14686996.2017.1316422)
Supplement: Suppl.pdf [file tsta_a_1316422_sm0893.pdf]

Supplementary materials

FMR spectra measured in 10 nm YIG layer grown at 1000°C for in-plane (a) and out-of-plane (b) orientation of magnetic field are present in Fig. 1SM.

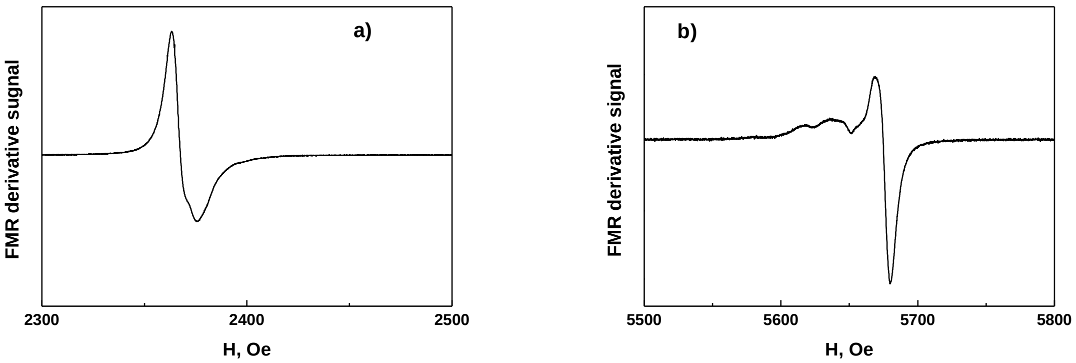

FIG. 1SM   Typical FMR specter of 10 nm YIG layer grown at 1000°C for in-plane (a) and out-of-plane (b) orientation of magnetic field.

The effective magnetization $4\pi M_s$-H_a in films was calculated from FMR spectra using the values of resonance magnetic fields in the in-plane (H_{in}) and perpendicular (H_{out}) orientations [5].

$$F_{||} = \gamma\left[H_{in}\left(H_{in} + 4\pi M_s - H_a\right)\right]^{1/2}, \qquad (1)$$

$$F_{\perp} = \gamma\left[H_{out} - \left(4\pi M_s - H_a\right)\right]. \qquad (2)$$

Here g is the gyromagnetic ratio; $F_{||}$ and $F_{\perp}$ are resonance frequencies for the in-plane and perpendicular magnetizations, respectively. The $4\pi M_s$-H_a and γ value is found by solving the equations (1), (2) with respect to variables γ and $4\pi M_s$-H_a and is presented in Table II. The line width ΔH is determined as the distance between the extrema of FMR signals. The line widths $\Delta H_{\perp}$ and $\Delta H_{||}$ for the perpendicular and in-plane orientations of the magnetic field are also shown in Table II.

Table   Number of structure, loop coercivity and in-plane anisotropy field, in-plane resonance field H_{in}, out-of-plane resonance field H_{out}, FMR line width ΔH_{in} and ΔH_{out}, calculated gyro-magnetic ratio γ, effective magnetic field H_{eff}.

Sample	H_C (Oe)	H_a (Oe)	H_{in} (Oe)	ΔH_{in} (Oe)	H_{out} (Oe)	ΔH_{out} (Oe)	γ (MHz/Oe)	H_{eff} (Oe)
8035	0.23	-1.3	2391	13	5601	14	2.82	2265
8037	0.4	-2.0	2510	12	5218	19	2.83	1897
8040	0.7	-2.7	2387	17	5607	25	2.82	2274
8043	0.8	~-2	2348	16	5769	22	2.81	2422
8044	0.35	-2.0	2367	11	5674	11	2.83	2350
